# ER and HER2 expression are positively correlated in HER2 non-overexpressing breast cancer

**DOI:** 10.1186/bcr3145

**Published:** 2012-03-14

**Authors:** Isabel Pinhel, Margaret Hills, Suzanne Drury, Janine Salter, Georges Sumo, Roger A'Hern, Judith M Bliss, Ivana Sestak, Jack Cuzick, Peter Barrett-Lee, Adrian Harris, Mitch Dowsett

**Affiliations:** 1 Academic Department of Biochemistry, The Institute of Cancer Research, Fulham Road, London, SW3 6JJ, UK; 2 Academic Department of Biochemistry, The Royal Marsden Hospital, Fulham Road, London, SW3 6JJ, UK; 3 Academic Department of Biochemistry, The Breakthrough Breast Cancer Research Centre, Fulham Road, London, SW3 6JJ, UK; 4 Clinical Trials and Statistics Unit, The Institute of Cancer Research, Cotswold Road, Sutton, SM2 5NG, UK; 5 Centre for Cancer Prevention, Wolfson Institute of Preventive Medicine, Queen Mary University of London, Charterhouse Square, London, EC1M 6BQ, UK; 6 Academic Breast Unit, Velindre Cancer Center, Velindre NHS Trust, Cardiff, CF14 2TL, UK; 7 Cancer Research UK Molecular Oncology Laboratories, Weatherall Institute of Molecular Medicine and Nuffield Department of Clinical Laboratory Sciences, John Radcliffe Hospital, OX3 9DS, Oxford, UK

## Abstract

**Introduction:**

Estrogen receptor-α (ER) and human epidermal growth factor receptor 2 (HER2) positivity are inversely correlated by standard criteria. However, we investigated the quantitative relation between ER and HER2 expression at both RNA and protein levels in HER2+ve and HER2-ve breast carcinomas.

**Methods:**

ER and HER2 levels were assessed with immunohistochemistry (IHC) and (for HER2) fluorescent *in situ *hybridization (FISH) and by quantitative reverse transcription-polymerase chain reaction (q-RT-PCR) in formalin-fixed primary breast cancers from 448 patients in the National Cancer Research Institute (NCRI) Adjuvant Breast Cancer Trial (ABC) tamoxifen-only arm. Relations at the RNA level were assessed in 1,139 TransATAC tumors.

**Results:**

ER and HER2 RNA levels were negatively correlated as expected in HER2+ve (IHC 3+ and/or FISH-amplified) tumors (*r *= -0.45; *P *= 0.0028). However, in HER2-ve tumors (ER+ve and ER-ve combined), a significant positive correlation was found (*r *= 0.43; *P *< 0.0001), HER2 RNA levels being 1.74-fold higher in ER+ve versus ER-ve tumors. This correlation was maintained in the ER+veHER2-ve subgroup (*r *= 0.24; *P *= 0.0023) and confirmed in this subgroup in 1,139 TransATAC tumours (*r *= 0.25; *P *< 0.0001). The positive relation extended to IHC-detected ER in ABC: mean ± 95% confidence interval (CI) H-scores were 90 ± 19 and 134 ± 19 for 0 and 1+ HER2 IHC categories, respectively (*P *= 0.0013). A trend toward lower relapse-free survival (RFS) was observed in patients with the lowest levels of ER and HER2 RNA levels within the ER+veHER2-ve subgroup both for ABC and TransATAC cohorts.

**Conclusions:**

ER and HER2 expression is positively correlated in HER2-ve tumors. The distinction between HER2+ve and HER2-ve is greater in ER-ve than in ER+ve tumors. These findings are important to consider in clinical trials of anti-HER2 and anti-endocrine therapy in HER2-ve disease.

**Trial Registration:**

Clinical trial identifier: ISRCTN31514446.

## Introduction

Estrogen receptor-α (ER) and the human epidermal growth factor receptor 2 (HER2) are the two key biomarkers that segregate the most distinct biologic subgroups of breast cancer and presently direct adjuvant treatment of primary disease. ER is expressed in approximately 80% of all breast cancers [1]. Amplification or overexpression of *HER2 *or both are present in about 15% of breast cancers [2,3]. The importance of these receptors as predictive biomarkers has been underpinned by the American Society of Clinical Oncology/College of American Pathologists (ASCO/CAP), who have issued guidelines for their accurate testing [4,5].

In HER2-overexpressing (HER2+ve) metastatic cancer, treatment with trastuzumab in combination with chemotherapy improves time to progression and survival [6]. In the adjuvant setting, sequential or concurrent trastuzumab improves disease-free survival and, in some trials, overall survival [7-9]. Despite its recognized benefit in HER2+ve disease, some evidence suggests this benefit also extends to HER2-non-overexpressing/normal (HER2-ve) cancer. Paik and colleagues [10] showed that patients with normal HER2 copy number and protein levels lower than 3+ by immunohistochemistry (IHC) may also benefit from trastuzumab. The NSABP B-47 (NCT 01275677) trial is now recruiting women with node-positive or high-risk node-negative HER2-low (IHC 1+ or IHC 2+ FISH-ve) invasive breast cancer to address these observations prospectively.

HER2 overexpression is associated with partial resistance to endocrine treatment [11-15]. The complex cross-talk between ER and HER2 pathways might be an underlying cause of resistance, although the intrinsic biologic mechanism is poorly understood [16,17]. Some aspects of the relation between ER and HER2 expression have been described previously. ER and HER2 positivity are inversely correlated [18-20], leading to approximately 10% of ER+ve tumors being HER2+ve [21] and about 50% of HER2+ve being ER+ve [22]. ER expression has also been shown to be quantitatively higher in HER2-ve than in HER2+ve tumors among ER+ve tumors; however, few analyses have been conducted on the quantitative relation within the HER2-ve subgroup [23]. In addition, little evidence exists for the biologic importance of IHC 1+ versus 0 levels of HER2 expression, with both categories currently classified as HER2-ve. It is unclear whether IHC 1+ cases arise as an artifact of staining or reflect a true difference in expression [10]. Furthermore, few data examine the relation between transcript levels and protein expression by IHC for HER2, even though this relation is well documented for ER [24].

We have therefore (a) characterized gene expression, protein, and/or amplification levels for ER and HER2 individually; (b) explored the relation between ER and HER2 at both the protein and RNA levels, irrespective of the samples ER and HER2 status; and (c) evaluated the significance of transcript levels within the HER2-ve population and, in particular, the ER+veHER2-ve/subgroup in terms of relapse-free survival in a set of 448 early breast cancers from the NCRI Adjuvant Breast Cancer (ABC) Trial-Tamoxifen Late Relapse Study [25,26].

## Materials and methods

### Sample collection

The ABC Trial was a randomized, controlled phase III clinical trial comparing the addition of (a) chemotherapy (with or without elective ovarian ablation or suppression), and (b) ovarian ablation or suppression (with or without elective chemotherapy) to prolonged treatment with tamoxifen in women with early-stage breast cancer [25,26]. All patients were accrued between 1992 and 2000 and received at least 5 years of tamoxifen (20 mg/day), irrespective of ER status. The ABC Tamoxifen Late Relapse (TLR) study was designed as a retrospective translational research study within the main ABC trial to identify predictive biomarkers of late relapse. Four hundred and forty-eight archival formalin-fixed paraffin-embedded (FFPE) tumor blocks were obtained from participating centers for patients from the tamoxifen-alone arm. This study was approved by the Multicentre Research Ethics Committee, and all patients included gave informed consent. Results on the primary aims of the ABC TLR study will be published separately.

### Immunohistochemistry and fluorescence *in situ *hybridization

Tissue microarrays (TMAs) were constructed by using single cores (1-mm diameter) taken from donor blocks containing sufficient invasive tumor. Serial 4-μm sections from the TMAs were used for immunohistochemical and fluorescence *in situ *hybridization (FISH) analyses.

ER was measured with immunohistochemistry (IHC) by using clone 6F11 (dilution 1/40; Leica Microsystems, Newcastle Upon Tyne, UK) and quantified with H-score [27]. ER positivity was defined as H-score ≥ 1, which corresponds closely to an Allred score ≥ 3 and to the recently recommended IHC positivity cut-off, according to ASCO/CAP guidelines of > 1% positive cells [4].

HER2 protein levels were assessed with the HercepTest (Dako Cytomation, Carpinteria, CA, USA) and considered positive if IHC staining was 3+, equivocal for IHC 2+, and negative for IHC 0 and 1+, as per ASCO/CAP guidelines [5]. HER2 amplification was measured with FISH by using the Pathvysion HER-2 DNA Probe kit (Abbott Molecular, Inc., Des Plaines, IL, USA) and considered positive, equivocal, or negative if FISH ratios (*HER2*/*CEP17*) were > 2.2, 1.8 to 2.2, or < 1.8, respectively [5]. For all markers, only the invasive tumor component was assessed.

### Quantitative reverse transcription-PCR

RNA extraction was performed on two 10-μm sections taken from each of the tumor blocks by using the RNeasy FFPE RNA Isolation kit (Qiagen, Crawley, UK), according to the manufacturer's recommendations, except for an initial incubation in xylene (30 minutes, 37°C) for complete removal of paraffin. RNA was additionally treated to remove genomic DNA contamination (1 hour, 37°C) with 6 U of rDNase I (DNA-*free *Kit; Applied Biosystems, Foster City, CA, USA). RNA quality and quantity were evaluated by using the Agilent 2100 Bioanalyser (Expert Software version B.02.03; Agilent Technologies, Edinburgh, UK).

Four hundred nanograms of RNA (in triplicate) was reverse transcribed with SuperScript III by using random primers (Invitrogen, Paisley, UK). Twenty nanograms of cDNA was analyzed with quantitative reverse transcription-PCR (qRT-PCR) in triplicate by using the ABI Prism 7900HT (Applied Biosystems). Reference genes consisted of *MRPL19*, *TFRC*, and *TBP*, as previously described [28]. The gene-expression assays and primers/probes used are detailed in Additional file 1, Table S1. ER and HER2 transcript data were normalized to the geometric mean of three reference genes (*MRPL19, TFRC*, and *TBP*) [29].

### Statistical analysis

ER and HER2 RNA data were log_10 _transformed. Spearman rank correlation was used to determine the correlation between protein and RNA levels for both ER and HER2 and between FISH ratio and RNA levels for HER2 only. Differences between subpopulation means were assessed with the Mann-Whitney test, and analysis of variance for multigroup comparisons used the Kruskal-Wallis test with GraphPad Prism 5.0a software (La Jolla, CA, USA).

Analysis of time-to-event data was undertaken by using Cox regression, multivariable fractional polynomials being used to investigate nonlinear relations and interactions between outcome and continuous parameters for ER and HER2 RNA levels [30] and with STATA 10.1 software (College Station, TX, USA). Transcript levels for ER and HER2 by using the Oncotype DX test were available for 1,139 HER2-ve tumors from the TransATAC study [31,32] for validation of results presented here. A *post hoc *hazard ratio test was used to detect differences in outcome between subgroups. All tests were two-sided and considered significant for *P *values < 0.05.

## Results

### Samples available

**Figure 1:**
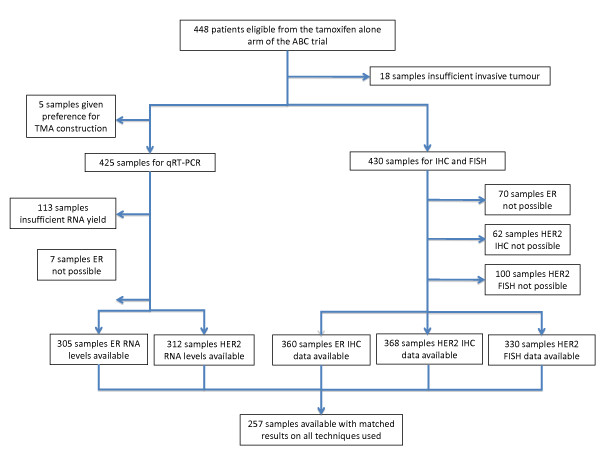
**CONSORT flow chart of the ABC Tamoxifen Late Relapse Study in which the number of samples with data available for ER and HER2 by qRT-PCR, IHC, and FISH (for HER2 only) are shown**. ER, estrogen receptor; FISH, fluorescence *in situ *hybridization; HER2, human epidermal growth factor receptor 2; IHC, immunohistochemistry; qRT-PCR, quantitative reverse-transcription polymerase chain reaction.

A complete set of results for ER and HER2 using qRT-PCR, IHC, and FISH (for HER2 only) was available for 257 samples, with the exception of 25 cases in which HER2 was available by only either IHC or FISH. Reasons for nonavailability for each marker are shown in the CONSORT diagram (Figure 1). HER2 IHC 2+ cases in which no FISH data were available were excluded from analysis where relevant (*n *= 4). All data presented are in relation to these matched results. The demographic characteristics of this population are shown in Table 1. Median follow-up was 8.1 years (interquartile range (IQR) = 5.2 to 10.3 years).

**Table 1:** Demographic characteristics of patients included in the ABC Tamoxifen Late Relapse Study

Variable	*N *= 257
Age in years, mean ± SD	54.8 ± 8.0
Tumor size, *n *(%)	
< 2 cm	122 (48.4)
2-5 cm	123 (48.8)
> 5 cm	7 (2.8)
Grade, *n *(%)	
Well differentiated	22 (8.6)
Moderately differentiated	110 (42.8)
Poorly differentiated	107 (41.3)
Unknown differentiated	18 (7.0)
Nodal status, *n *(%)	
Negative	100 (40.8)
1 to 3 nodes positive	106 (43.3)
4+ positive	39 (15.9)
Local ER status, *n *(%)	
Positive	133 (51.7)
Negative	84 (32.7)
Unknown	40 (15.6)

A complete set of results for ER and HER2 by using qRT-PCR, IHC, and FISH (for HER2 only) was available. ER, estrogen receptor; SD, standard deviation.

### ER- and HER2-positive/negative status by IHC and FISH

Sixty-seven percent of cases were ER+ve (*n *= 173), and the remaining 33% were negative (*n *= 84). HER2 by IHC was positive for 15% (IHC 3+; *n *= 37), equivocal for 8% (IHC 2+; *n *= 19), and negative for 77% of cases (IHC 0, 38%; *n *= 96; 1+, 39%, *n *= 101). HER2 was amplified in 15% (*n *= 34) and remained equivocal in 1% of cases (*n *= 3), which could not be assessed in 20 further cells because of scarce invasive tumor. Overall, HER2 was positive by either IHC or FISH in 16% of cases (*n *= 42). HER2 distribution by IHC and FISH according to positive/negative status is shown in Table 2.

**Table 2:** HER2 distribution by IHC and FISH

HER2		FISH				
		
		Positive	Negative	Equivocal	Not available	Total
**IHC**	Positive (3+)	29	5	2	1	37
	Equivocal (2+)	2	13	-	4	19
	Negative (1+)	2	91	1	7	101
	Negative (0)	1	86	-	9	96
	Not available	-	4	-	-	4
	Total	34	199	3	21	257

FISH, fluorescence *in situ *hybridization; HER2, human epidermal growth factor receptor 2; IHC, immunohistochemistry.

### Correlation between RNA and IHC (FISH) levels for ER and HER2

The measurement of ER and HER2 transcript levels by qRT-PCR in FFPE tissue was previously validated in our laboratory [28].

**Figure 2:**
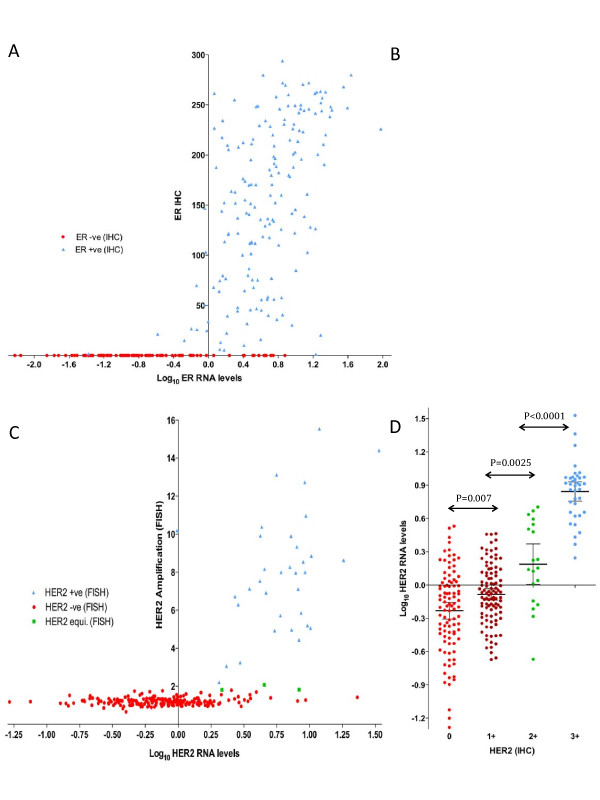
**Relation of RNA levels**. **(a) **Relation of ER RNA levels (qRT-PCR) with protein expression assessed with IHC (H score). **(b) **ER RNA levels according to IHC status. ER-ve (IHC H score ≤ 1); *n *= 84; and ER+ve (IHC H score > 1); *n *= 173. **(c) **Relation of HER2 RNA levels (qRT-PCR) with HER2 amplification levels (FISH). HER2+ve, *n *= 34; HER2-ve, *n *= 199; and HER2 equivocal, *n *= 3. **(d) **HER2 RNA levels according to IHC HercepTest categories. IHC 0, *n *= 96; IHC 1+, *n *= 101; IHC 2+, *n *= 19; and IHC 3+, *n *= 37. Means with 95%CI are shown. CI, confidence interval; ER, estrogen receptor; FISH, fluorescence *in situ *hybridization; HER2, human epidermal growth factor receptor 2; IHC, immunohistochemistry; qRT-PCR, quantitative reverse-transcription polymerase chain reaction.

ER was highly correlated at the RNA and protein levels (Figure 2a; Spearman *R *= 0.76; *P *< 0.0001). This correlation was less strong but still highly significant in the ER+ve population only (blue symbol, Figure 2a; Spearman *R *= 0.53; *P *< 0.0001). ER RNA levels were a mean 9.52-fold higher in IHC-positive than in IHC-negative tumors, although levels between the two categories overlapped (Figure 2b; *P *< 0.0001).

HER2 transcript and amplification levels were significantly correlated (Figure 2c; Spearman *R *= 0.41; *P *< 0.0001), and this correlation was also found to extend to the FISH-positive (FISH+ve) population only, albeit not so strongly (blue symbol, Figure 2c; Spearman *R *= 0.34; *P *= 0.047). HER2 transcript levels were a mean 6.52-fold higher in FISH+ve than in FISH-negative (FISH-ve) tumors (*P *< 0.0001). Figure 2d shows HER2 RNA levels according to each of the IHC categories (0, 1+, 2+, and 3+). Despite substantial overlap between the groups, a multigroup comparison showed a significant difference between the four categories (*P *< 0.0001). HER2 RNA levels were significantly different between each of the adjacent IHC categories: 0 versus 1+, *P *= 0.0070; 1+ versus 2+, *P *= 0.0025; 2+ versus 3+, *P *< 0.0001.

### Relation of ER with *HER2 *gene expression

The relation of ER with HER2 at the gene-expression level was investigated separately in HER2+ve (FISH+ve and/or IHC 3+; *n *= 42) and HER2-ve cases (FISH-ve and IHC 0/1+/2+; *n *= 211). Four IHC 2+ cases were further excluded, for which FISH data were unavailable for confirmation. The analyses included both ER+ve and ER-ve cases.

**Figure 3:**
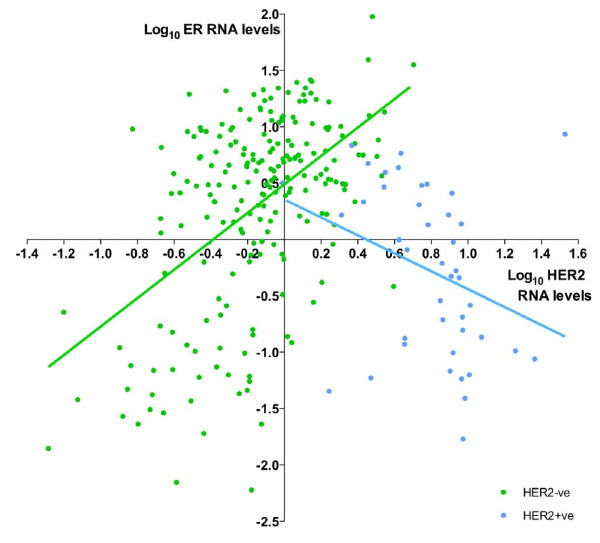
**Correlation of ER and HER2 transcript levels (qRT-PCR)**. HER2-ve is defined as HER2 FISH-ve and IHC 0/1+/2+; *n *= 211. HER2+ve is defined as HER2 FISH+ve and/or IHC 3+; *n *= 42. ER, estrogen receptor; FISH, fluorescence *in situ *hybridization; HER2, human epidermal growth factor receptor 2; IHC, immunohistochemistry; qRT-PCR, quantitative reverse-transcription polymerase chain reaction.

The expected significant inverse correlation between ER and HER2 RNA levels in the HER2+ve group was observed (Spearman *R *= -0.45; *P *= 0.0028; Pearson *R *= -0.32; *P *= 0.041; Figure 3). In contrast, a significant and equally strong positive correlation was found between ER and HER2 RNA levels in the HER2-ve group (Spearman *R *= 0.43; *P *< 0.0001; Pearson *R *= 0.51; *P *< 0.0001; Figure 3).

**Figure 4:**
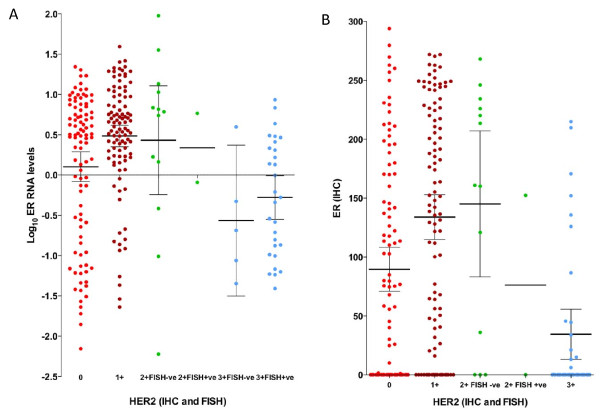
**RNA and protein levels**. **(a) **ER RNA levels and **(b) **ER protein levels according to HER2 protein and gene-amplification levels (IHC HercepTest and FISH, respectively); IHC 0, *n *= 96; IHC 1+, *n *= 101; IHC 2+ FISH-ve, *n *= 13; IHC 2+ FISH+ve, *n *= 2; IHC 3+ FISH-ve, *n *= 5; IHC 3+ FISH+ve, *n *= 29; IHC 3+, *n *= 37. Means with 95% CI are shown (95% confidence intervals for IHC2+ FISH+ve subgroup are **4a**, -5.1 to 5.8, and **4b**, -892.2 to 1045; these were omitted for ease of visualization). CI, confidence interval; ER, estrogen receptor; FISH, fluorescence *in situ *hybridization; HER2, human epidermal growth factor receptor 2; IHC, immunohistochemistry; qRT-PCR, quantitative reverse-transcription polymerase chain reaction.

The direct relation between ER and HER2 transcript levels in HER2-ve tumors was also found in the relation between ER RNA levels and HER2 at the protein level (Figure 4a). ER RNA levels in the six HER2 categories were significantly different by multigroup comparison (*P *< 0.0001), with ER RNA levels being higher in the HER2 IHC 1+ and 2+FISH-ve group than in the IHC 0 group (*P *= 0.01 and *P *= 0.15, respectively). ER RNA levels in each of the HER2 IHC categories 0, 1+, and 2+ were nonetheless significantly higher than in IHC 3+ FISH+ve tumors (*P *= 0.0095, *P *< 0.0001, and *P *= 0.011, respectively).

In ER+ve disease, ER RNA levels were a mean 2.73-fold higher in HER2-ve than in HER2+ve tumors (8.21 versus 3.01; *P *= 0.0014; Table 3; Additional file 2: Figure S1a). In HER2-ve tumors, the RNA level of HER2 was higher in ER+ve than in ER-ve tumors, with a mean fold difference of 1.75 (1.09 versus 0.62; *P *< 0.0001; Additional file 2: Figure S1b). In ER+ve disease, HER2 RNA levels were 6.08-fold higher in HER2+ve than in HER2-ve tumors (6.63 versus 1.09; *P *< 0.0001). This difference was more evident in the ER-ve population, in which a 13.7-fold difference was found between HER2+ve and HER2-ve tumors (8.51 versus 0.62; *P *< 0.0001).

**Table 3:** Number (%) of samples within the positive and negative subgroups for ER and HER2 biomarkers

Subgroups	Number (%)	ER RNA levels^a^	HER2 RNA levels^a^
ER+HER2-ve	155 (60)	8.21 (6.59-9.83)	1.09 (0.96-1.21)
ER+HER2+ve	18 (7)	3.01 (1.89-4.13)	6.63 (3.01-10.2)
ER-HER2-ve	60 (23)	0.93 (0.48-1.37)	0.62 (0.47-0.77)
ER-HER2+ve	24 (9)	0.50 (9.04 × 10^-2 ^- 0.92)	8.51 (6.60-10.4)

**^a^**Mean (95% CI) values are shown. ER+ve, IHC H-score ≥ 1; HER2-ve, FISH-ve and IHC 0/1+/2+; HER2+ve, FISH+ve and/or IHC 3+. CI, confidence interval; ER, estrogen receptor; FISH, fluorescence *in situ *hybridization; HER2, human epidermal growth factor receptor 2; IHC, immunohistochemistry.

The positive correlation observed between ER and HER2 transcript levels in HER2-ve samples was also maintained, albeit more weakly when that group was further divided to include ER+ve tumors only (Spearman *R *= 0.24; *P *= 0.0023). This finding was present to a similar degree in this ER+ve/HER2-ve subgroup in TransATAC (Spearman *R *= 0.25; *P *< 0.00001). ER H-scores were significantly higher in HER2 IHC 1+ than in IHC 0 tumors (mean ± SEM, 134 ± 10 and 90 ± 9, respectively; *P *= 0.0013; Figure 4b), suggesting further evidence for a direct relation between the two biomarkers in HER2-ve disease.

### Prognostic relevance of ER and HER2 RNA levels

**Figure 5:**
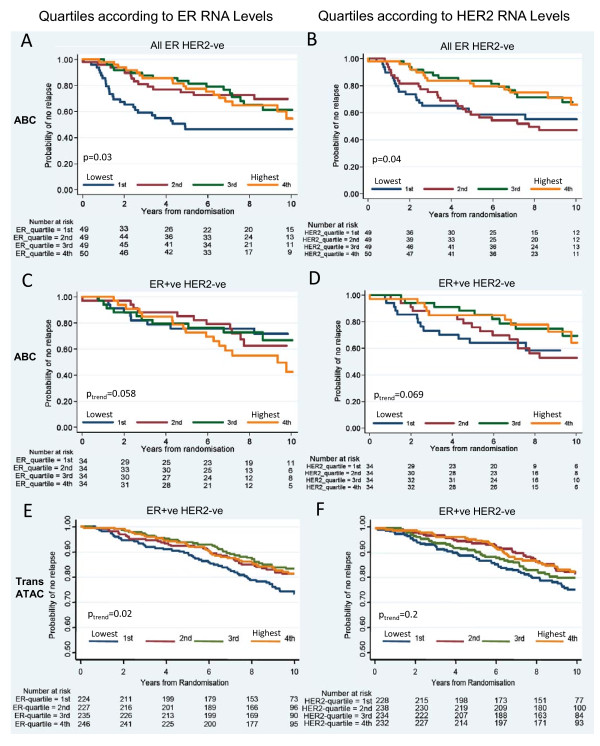
**Kaplan-Meier plots for relapse-free survival in HER2-ve cases**. **(a, b) **Relapse-free survival by ER and HER2 RNA quartiles (qRT-PCR), respectively, in the HER2-ve population of the ABC study, irrespective of ER status. **(c, d) **Relapse-free survival by ER and HER2 RNA quartiles, respectively, in the subgroup of ER+ve HER2-ve cases of the ABC study. **(e, f) **Relapse-free survival by ER and HER2 RNA quartiles, respectively, in the ER+ve HER2-ve cohort of the TransATAC study. First quartile defined as the lowest RNA levels for ER and HER2. N.B.: The y-axis scale in the ABC study is different from that in the TransATAC study. ER, estrogen receptor; HER2, human epidermal growth factor receptor 2; qRT-PCR, quantitative reverse-transcription polymerase chain reaction.

We explored the potential clinical importance of ER and HER2 expression in the HER2-ve cases (*n *= 197). The log-rank test showed a significant difference in time to relapse (TTR) between groups, defined by quartiles of ER RNA levels (*P *= 0.03), with shortest TTR in those with the lowest ER quartile, which included most of the ER-ve cases (Figure 5a). TTR was also significantly different according to HER2 levels (*P *= 0.04), being higher in the 50% of patients with the lowest HER2 RNA levels (Figure 5b).

Considering only the ER+ve HER2-ve cases, a test for trend showed a nonsignificant trend to a difference in TTR between the quartiles of RNA levels for ER and HER2 (Figure 5c, p*_trend _*= 0.058; and Figure 5d, p*_trend _*= 0.069, respectively). Comparing the lowest quartile of HER2 RNA levels with the others in that subgroup, a significant difference in TTR at 5 years was found; this became nonsignificant at 10 years (HR (95% CI), 2.45 (1.17 to 5.12) and 1.53 (0.82 to 2.86), respectively).

In the TransATAC study, a similar pattern was observed in the ER+ve HER2-ve cohort. A significant difference in TTR between quartiles of ER RNA levels was found (Figure 5e, p*_trend _*= 0.02), with the lowest quartile showing the shortest TTR, which was not consistently observed throughout the 10-year follow-up in ABC, possibly because of the small number of cases. With regard to HER2 RNA levels, a nonsignificant trend to a difference in TTR between quartiles was observed (Figure 5f; p*_trend _*= 0.2). The lowest quartile of HER2 levels showed a trend to shortest TTR at 5 and 10 years (HR (95% CI), 0.83 (0.51 to 1.36) and 0.76 (0.55 to 1.06), respectively). A significant relation between ER protein levels and outcome in TransATAC has been published [21].

## Discussion

To our knowledge, the present study is the first to report a direct relation between ER and HER2 at the RNA level in HER2-ve tumors. The TransATAC set of material provided confirmation of this observation, and two other studies support this relation at the protein level but do not examine it in detail [23,33].

ER and HER2 status is used to guide the decision-making process for patient treatment, but clinical trials have been conducted (for example, letrozole ± lapatinib, EGF30008 [34]) and are under way (chemotherapy ± trastuzumab, NSABP-B47) of HER2-targeted agents in HER2-ve disease in which the relations between (a) ER and HER2 and (b) HER2 IHC and mRNA expression are not well described. The present study revealed novel data that, as well as being of general biologic interest, may be important to the interpretation or conduct of these trials, respectively. In addition, the higher HER2 RNA expression in the ER+ve HER2-ve tumors might provide an escape route for such tumors during endocrine therapy.

Here, ER and HER2 gene expression were measured on FFPE material with qRT-PCR. This technique allows a quantitative evaluation of biomarker expression and generation of data as a continuous variable, as opposed to IHC, which, other than by use of specialist techniques such as AQUA [35], at most provides a semiquantitative reading, with categorization of data potentially leading to loss of information. Previous data from our laboratory had demonstrated an excellent correlation between fresh-frozen and FFPE materials for both ER and HER2 [28]. The reliability of gene expression measured with qRT-PCR in FFPE material, including ER and HER2 expression, has been extensively validated [24] and is now included in commercial prognostic tools, such as Oncotype DX [36].

The well-described inverse relation between ER and HER2 based on status and the negative quantitative relation between ER and HER2 expression in HER2+ve tumors was confirmed in our study [18-20]. Given this, the positive correlation between the RNA levels of ER and HER2 for HER2-ve patients was surprising but was confirmed as being present at a protein (IHC) level. The validity of the direct relation between ER and HER2 RNA levels was confirmed in the large TransATAC series of ER+veHER2-ve tumors. Data in the literature support this relation. Konecny *et al. *[23] reported, but did not characterize further, a significant positive correlation between these markers at the protein level among HER2-ve tumors within their cohort A. Harigopal *et al. *[33] also described a positive correlation between ER and HER2 detected with AQUA in tumors expressing ER and HER2 at the lowest quartiles of expression. In addition, the bottom (low) as well as the top (high) deciles of HER2 expression were significantly associated with worse disease-free survival. Our results also show worse relapse-free survival for the HER2-ve tumors expressing HER2 at the lowest levels. This relation with outcome was relatively modest but was consistent with the validation analyses undertaken with the TransATAC cohort. The significantly worse outcome shown for the lowest ER quartile probably reflects that this group is largely ER-ve; this effect was lost when the analysis was confined to ER+ve tumors.

Currently, HER2 diagnostics for selecting patients for trastuzumab or other HER2-targeted treatments are based on a dichotomous categorization whereby patients with FISH+ve or IHC 3+/2+FISH+ve tumors are deemed positive. The data presented here reveal widely overlapping transcript levels between these conventionally HER2-positive and -negative categories, as well as between the "negative" IHC subcategories of 0, 1+, and 2+FISH-ve. Brase *et al. *[37] and Paik *et al. *[10] also showed a continuous expression of HER2 RNA levels according to IHC/FISH categories. It is notable that although a major overlap exists between the groups, the HER2 IHC 1+ category shows significantly higher RNA levels than the IHC 0 category (Figure 2d), suggesting that some biologic meaning is associated with the separation of these IHC staining groups, rather than being merely a staining artefact. Although its importance is at present uncertain, Paik and colleagues [10] found HER2-ve patients to benefit from trastuzumab to a similar extent as do HER2+ve patients [10]. This finding will be prospectively addressed in the recently initiated NSABP-B47 study of trastuzumab in HER2 IHC 1+ or IHC 2+ FISH-ve cases (NCT 01275677). Our results indicate that, at least at a transcript level, these groups are only modestly different from HER2 IHC 0 cases. An assessment of the quantitative level of HER2 at the transcript level is merited, in that and other studies of this population, as a possible predictor of benefit. This continuous expression of HER2 at a transcript level, as noted earlier, is also seen with techniques such as AQUA and may have implications for the targeting of agents such as the HER2-cytotoxic conjugate TDM-1, which may rely more on the level of binding to cells than an opposing underlying HER2-driven growth [38].

Our data also have implications for the confidence in ascribing of HER2 positivity in diagnostics. The difference in HER2 RNA levels between HER2+ve and HER2-ve tumors was more evident in the ER-ve than in the ER+ve population (13.7-fold versus 6.1-fold difference, respectively). These data suggest that the distinction of HER2+ve from HER2-ve tumors in pathologic diagnosis may be subject to greater error in ER+ve than in ER-ve patients.

Our study has some limitations. Single-core TMAs were used for ER and HER2 IHC assessment, and although the expression of these markers is not highly heterogeneous, some relations may be weakened. It is unlikely, though, that this might lead to false-positive relations. These IHC/FISH TMA results were compared with qRT-PCR results from whole sections, and this is also likely to add to the variability observed. However, given that a false-positive finding has been excluded by the TransATAC validation dataset, this variability is most likely to reduce the strength of the reported relations.

## Conclusions

ER and HER2 are positively correlated at both protein and RNA levels in HER2-ve tumors, contrary to their recognized negative relation in HER2+ve disease. The distinction between HER2+ve and HER2-ve is greater in ER-ve than in ER+ve tumors. These findings may lead to greater diagnostic uncertainties in ER+ve patients and are important to consider in clinical trials of anti-HER2 and anti-endocrine therapy in HER2-ve disease.

## Abbreviations

ABC: NCRI Adjuvant Breast Cancer Trial; AQUA: automated quantitative analysis; ASCO: American Society of Clinical Oncology; CAP: College of American Pathologists; cDNA: complementary DNA; CEP17: centromere enumeration probe for chromosome 17; DNA: deoxyribonucleic acid; ER: estrogen receptor-α; FFPE: formalin-fixed paraffin-embedded; FISH: fluorescence *in situ *hybridization; HER2: human epidermal growth factor receptor 2; HR: hormone receptor; IHC: immunohistochemistry; MRPL19: mitochondrial ribosomal protein L19; NCRI: National Cancer Research Institute; NSABP: National Surgical Adjuvant Breast and Bowel Project; qRT-PCR: quantitative reverse-transcription polymerase chain reaction; RFS: relapse-free survival; RNA: ribonucleic acid; TMA: tissue microarray; TBP: TATA-binding protein; TFRC: transferrin receptor; TransATAC: translational anastrozole (Arimidex): tamoxifen: alone or in combination.

## Competing interests

All authors declare no conflicts of interest. JC and IS declare funding from AstraZeneca for continued follow-up of the ATAC trial by the Wolfson Institute of Preventive Medicine.

## Authors' contributions

IP contributed to the design of the study, conducted qRT-PCR analysis for ER and HER2, IHC staining and scoring for ER and HER2, statistical analysis, interpretation of data, and drafted the manuscript. MH carried out HER2 FISH analysis. SD established qRT-PCR assay quantification of ER and HER2. JS provided supervision of the immunohistochemical tests. GS and RA conducted survival analysis in the ABC study. RA also contributed to interpreting results and drafting the manuscript. IS and JC provided data from and conducted survival analyses in the TransATAC study. JB, PBL, and AH conceived and designed the study, provided study materials, and contributed to drafting the manuscript. MD conceived and designed the study and contributed to interpreting results and drafting the manuscript. All authors read and approved the final manuscript.

## Supplementary Material

Additional file 1 **Table S1: Details of gene-expression assays**. TaqMan Gene-expression assays and primers/probe used for quantification of genes of interest and reference genes by qRT-PCR (all purchased from Applied Biosystems). ER, estrogen receptor; HER2, human epidermal growth factor receptor 2; MRPL19, mitochondrial ribosomal protein L19; TBP, TATA box binding protein; TFRC, transferrin receptor protein 1. Click here for file

Additional file 2 **Figure S1: ER and HER2 RNA levels according to ER/HER2 subgroup**. ER and HER2 RNA levels (qRT-PCR) according to each of the subgroups ER+veHER2+ve, ER+veHER2-ve, ER-veHER2+ve, and ER-veHER2-ve. ER+ve defined as IHC H-score ≥ 1. HER2-ve defined as FISH-ve and IHC 0/1+/2+. HER2+ve defined as FISH+ve and/or IHC 3+. ER, estrogen receptor; HER2, human epidermal growth factor receptor 2; qRT-PCR, quantitative reverse-transcription polymerase chain reaction; IHC, immunohistochemistry; FISH, fluorescence *in situ *hybridization. Click here for file
