# Supplementary material for: ER and HER2 expression are positively correlated in HER2 non-overexpressing breast cancer
Source: Breast Cancer Res. 2012 Mar 14;14(2):R46. doi: 10.1186/bcr3145 (PMC3446380; doi:10.1186/bcr3145)
Supplement: Additional file 1 — Table S1: Details of gene-expression assays. TaqMan Gene-expression assays and primers/probe used for quantification of genes of interest and reference genes by qRT-PCR (all purchased from Applied Biosystems). ER, estrogen receptor; HER2, human epidermal growth factor receptor 2; MRPL19, mitochondrial ribosomal protein L19; TBP, TATA box binding protein; TFRC, transferrin receptor protein 1. [file bcr3145-S1.DOCX]

| Gene | Assay / Nucleotide sequence |
| --- | --- |
| ER | Hs01046818_m1 |
| HER2 | Hs01001580_m1 |
| MRPL19 | Hs00608522_g1 |
| TFRC | Hs00174609_m1 |
| TBP | Forward Primer: 5’- GCCCGAAACGCCGAATAT-3’; Reverse Primer: 5’- CGTGGCTCTCTTATCCTCATGA-3’;  Fluorescent probe TaqMan® TAMRA™: 6FAM-ATCCCAAGCGGT TTGCTGCGGTA-TAMRA |
