# Supplementary figures and images for: ER and HER2 expression are positively correlated in HER2 non-overexpressing breast cancer
Source: Breast Cancer Res. 2012 Mar 14;14(2):R46. doi: 10.1186/bcr3145 (PMC3446380; doi:10.1186/bcr3145)

## Slide 1
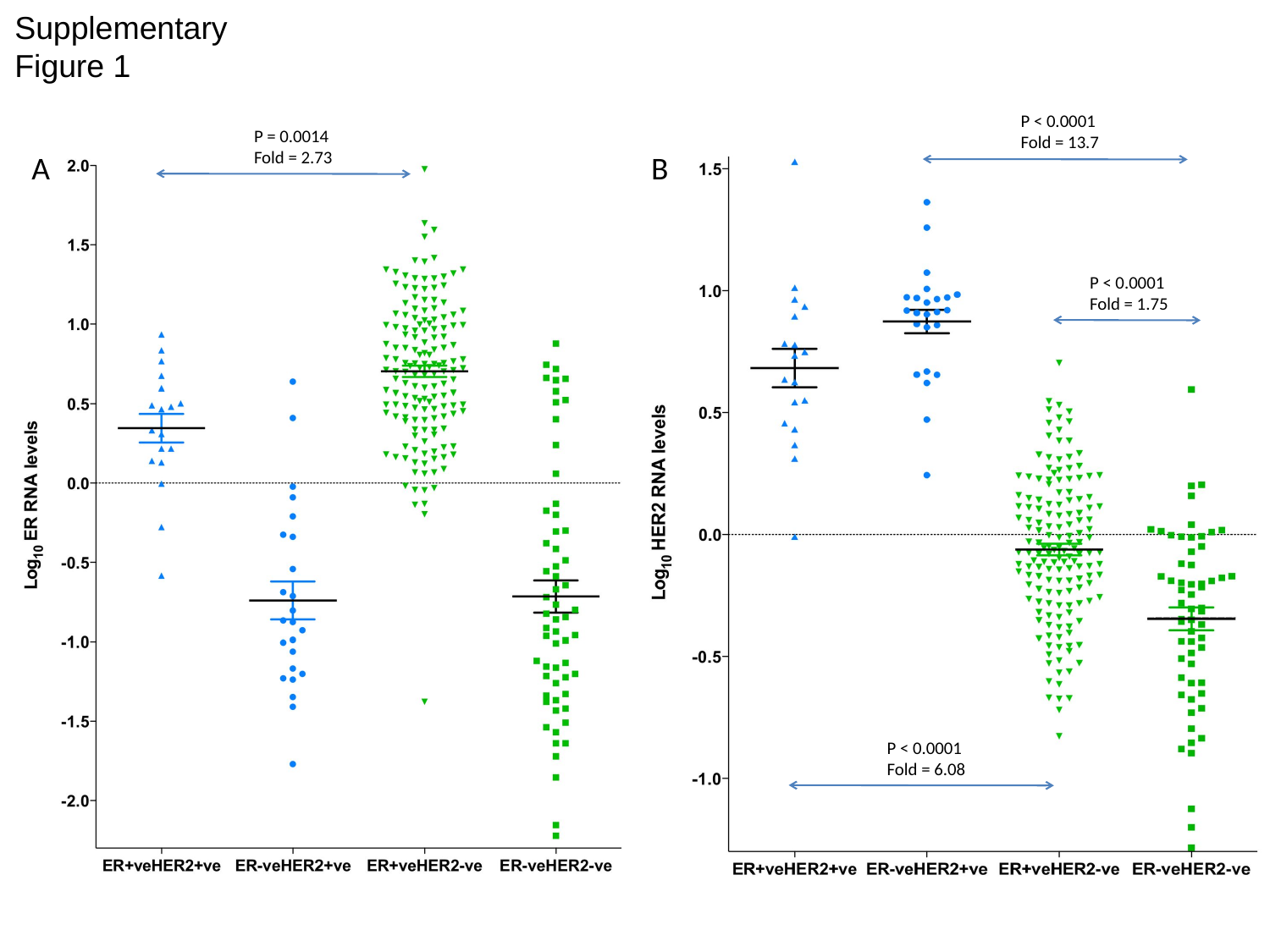

Supplementary
Figure 1
P < 0.0001
Fold = 13.7
P = 0.0014
Fold = 2.73
B
A
P < 0.0001
Fold = 1.75
P < 0.0001
Fold = 6.08

Supplement: Additional file 2 — Figure S1: ER and HER2 RNA levels according to ER/HER2 subgroup. ER and HER2 RNA levels (qRT-PCR) according to each of the subgroups ER+veHER2+ve, ER+veHER2-ve, ER-veHER2+ve, and ER-veHER2-ve. ER+ve defined as IHC H-score ≥ 1. HER2-ve defined as FISH-ve and IHC 0/1+/2+. HER2+ve defined as FISH+ve and/or IHC 3+. ER, estrogen receptor; HER2, human epidermal growth factor receptor 2; qRT-PCR, quantitative reverse-transcription polymerase chain reaction; IHC, immunohistochemistry; FISH, fluorescence in situ hybridization. [file bcr3145-S2.PPT]
